# Erectile dysfunction during receptive anal intercourse: an overlooked entity?

**DOI:** 10.1093/jsxmed/qdaf126

**Published:** 2025-06-29

**Authors:** Thomas W. Gaither, Myles Anderson, Jonathan Balcazar, Marcia Russell, Mark Litwin

**Affiliations:** 1 Department of Urology, David Geffen School of Medicine, University of California Los Angeles, Los Angeles, CA 90095, United States; 2 Burnett School of Medicine, Texas Christian University, Fort Worth, TX 76109, United States; 3 Department of Surgery, Section of Colorectal Surgery, David Geffen School of Medicine, Los Angeles 90095, United States; 4 Surgical and Perioperative Careline, VA Greater Los Angeles Healthcare System, Los Angeles, CA 90073, United States; 5 Department of Health Policy and Management, Fielding School of Public Health, University of California, Los Angeles, Los Angeles, CA 90095, United States; 6 School of Nursing, University of California Los Angeles, Los Angeles, CA 90095, United States

**Keywords:** erectile dysfunction, receptive anal intercourse, urology

## Introduction

Erectile dysfunction (ED) is commonly defined as the inability to achieve or maintain an erection sufficient for sexual intercourse or pleasure. Apart from the International Index of Erectile Function (IIEF), which has a modified scale for men who have sex with men and are HIV positive, ED has been exclusively studied in the context of penetrative intercourse.^1^ The Sexual Health Inventory for Men (SHIM) is a shortened form of the IIEF, which has two (out of five) questions specific to penetration. However, for males who practice receptive anal intercourse (RAI), the SHIM may not adequately capture their experience of ED. A previous qualitative study examining RAI among individuals with prostates found that while some participants may experience loss of erection, they still reported pleasure during RAI. Others indicated that maintaining an erection could help mitigate discomfort, with the loss of erection potentially reducing enjoyment. These findings suggest that the experience of ED during RAI may differ from traditional definitions.^2^ Supplementary Figure 1 includes some salient quotes from participants speaking about erections during RAI which conceptualized this construct. Based on the findings from this qualitative study, ED may have an alternate definition among those who practice RAI. Our study seeks to refine the definition of ED in the context of RAI and investigate its correlates.

Between July 2022 and January 2023, we conducted an online survey about sensations felt during RAI among people with prostates (ie, cisgender men, transgender women). Inclusion criteria for the survey included being assigned male at birth, having engaged in RAI within the past 6 months, being 18 years or older, and able to read English. All variables collected were self-reported. We used convenience sampling and snowball techniques to recruit participants via social media. The study protocol was approved by our Institutional Review Board.

As the current study focuses primarily on erectile function, only cisgender men were included. We measured erectile function during RAI via two questions on a 5-point Likert scale. The first question asked, “How often did you experience an erection while bottoming during the past six months?”. Possible responses ranged from never to always. The second question asked, “How big of a bother, if any, has losing an erection or not having one been while bottoming during the past six months?”. Possible responses ranged from “not bothersome OR I don’t experience this” to “extremely bothersome”. As no formal definition for ED during RAI exists, we defined ED as *sometimes*, *rarely*, or *never* experiencing an erection during RAI with *moderate*, *very*, or *extreme* bother (n = 213). The control group was the remainder of the participants.

As no gold standard definition for ED during RAI exists, we performed two sensitivity analyses to determine the robustness of our results. In our first sensitivity analysis (herein sensitivity analysis A) ED was defined as *rarely* or *never* experiencing an erection during RAI with *very or extreme* bother (n = 46). In our second sensitivity analysis (herein sensitivity analysis B), we varied the control group. As some participants in the primary analysis had poor erectile frequency but no associated bother, these participants may not be the best group to represent “erectile function.” Thus, this group was removed from the control group in the second sensitivity analysis (n = 86).

Exposure variables were selected a priori and selected based on previous literature and expert opinion. Exposure variables included age, sexual frequency, RAI lifetime exposure, sexualized drug use, mental health symptoms, urinary and bowel symptoms, erectile function, and pelvic sensations experiences during RAI.

For statistical analysis we used *t*-tests and Mann–Whitney *U* tests for parametric and non-parametric continuous data between those with ED and those without. All analyses were repeated for each of the sensitivity analyses. *P* values < .05 were considered statistically significant. All statistical analysis was performed in Stata v 17 (College Station, TX).

Our sample included 1040 cisgender male individuals. Basic demographics are outlined in Supplementary Table 1 within the supplemental material. The mean age of our participants was 36 (SD 12.6). Most participants identified as gay (82%). Out of our sample, 214 participants (21%) were classified as having ED during RAI based on our cut-off criteria, and 827 participants (79%) were classified as not having ED during RAI (Supplementary Table 2).

For the participants classified as having ED during RAI, the median SHIM score was 18 (interquartile range [IQR] = 13–22), which was significantly lower than the median score of 20 (IQR 16–24) for those who were classified as having no ED (*P* < .01). The participants in the upper quartile who met criteria by our definition for ED, however, still demonstrated a SHIM score that demonstrates no ED (> 20). Additionally, participants below the 50th percentile with no ED by our definition displayed a SHIM score < 21.

When compared to participants with ED, participants with no ED during RAI reported significantly higher pain levels (17 [IQR 13–21] vs 15 [IQR 12–19], *P* < .01), lower levels of pleasure (15 [IQR 12–17] vs 16 [IQR 14–18], *P* < .01), and higher levels of urinary (7 [IQR 5–9] vs 5 [IQR 4–7], *P* < .01) and bowel symptoms (9 [IQR 6–12] vs 7 [IQR 5–10], *P* < .01). Orgasm frequency was also significantly lower for participants with ED during RAI, 4 (IQR 3–4), compared to participants without ED, 4 (3–5) (*P* = .02). When assessing sexualized drug use, methamphetamine (1 [IQR 1–1] vs 1 [IQR 1–1]; mean 1.4 vs 1.1) and nicotine (1 [IQR 1–2] vs 1 [IQR 1–1]) use was significantly higher for those with ED during RAI (*P* < .01). There was no significant difference in the age of participants, sexual frequency, RAI lifetime exposure, benign prostatic hyperplasia symptoms, mental health symptoms, or chronic prostatitis symptoms (Supplementary Table 2).

The sensitivity analysis revealed consistent results, with ED during RAI continuing to be associated with lower pleasure scores, higher pain, urinary, and bowel symptoms, and increased use of methamphetamine and nicotine during sex (Supplementary Tables 3 and 4).

Erectile function in the context of RAI appears to have distinct aspects compared to penetrative erectile function. Our primary aim was to measure the prevalence of ED during RAI and assess possible associations with ED. The prevalence of ED during RAI varied from 4% of the sample using the stricter definition to 20%-22% using the less strict cut-off. Using a strict cut-off to determine ED during RAI may overlook individuals with milder symptoms, suggesting that ED in this context may be better understood as a spectrum rather than a dichotomous condition. Notably, approximately 25% of the sample with ED during RAI had normal SHIM scores. These findings suggest that existing erectile function measures, such as the SHIM, may not fully capture ED during RAI. Some data suggest that men may choose to engage in RAI because of erectile issues which prevent them from performing penetrative intercourse.^3^ Pelvic sensations, such as pain, urinary, or bowel symptoms and sexualized drug use, especially methamphetamine and nicotine, are associated with ED during RAI. Disrupting sensations during RAI may lead to detumescence either physically or psychologically. A previous survey found a relationship between sexual pain and problems with an erection, but the relationship was confounded by age, performance anxiety, and internalized homophobia.^4^ We acknowledge that alternative explanations, such as psychological factors (ie, performance anxiety), contextual variables (ie, alcohol use, partner dynamics), and undiagnosed conditions (ie, pelvic floor dysfunction) could also play a role. Further research is needed to better understand these relationships and determine whether targeted interventions could address ED during RAI.

This is a large cross-sectional study assessing a previously understudied sexual dysfunction. Limitations include lack of standard definition for ED during RAI, confounding, and lack of generalizability to individuals who do not complete Internet-based surveys. As a result, while we can identify correlations and associations, we cannot infer directionality or causality. The sample may also be skewed toward individuals with higher comfort or experience with online surveys, potentially limiting the generalizability of the findings to the broader population, especially those who do not engage with Internet-based research. While these findings highlight an area for further investigation, clinical validation studies will be needed before definitive conclusions can be drawn. However, recognizing ED in receptive contexts may call for a more tailored approach beyond standard protocols.

## Supplementary Material

Fig1

Table1

Table3

Table2

Table4

Supplementary material is available at *The Journal of Sexual Medicine* online.
