# Supplementary material for: Erectile dysfunction during receptive anal intercourse: an overlooked entity?
Source: J Sex Med. Author manuscript; Available in PMC 2026 Jan 14. (PMC12802361; doi:10.1093/jsxmed/qdaf126)
Supplement: Fig1 [file NIHMS2136089-supplement-Fig1.docx]

Figure 1. Brief conceptual framework around erectile function during receptive anal intercourse, data from 2020-2021*


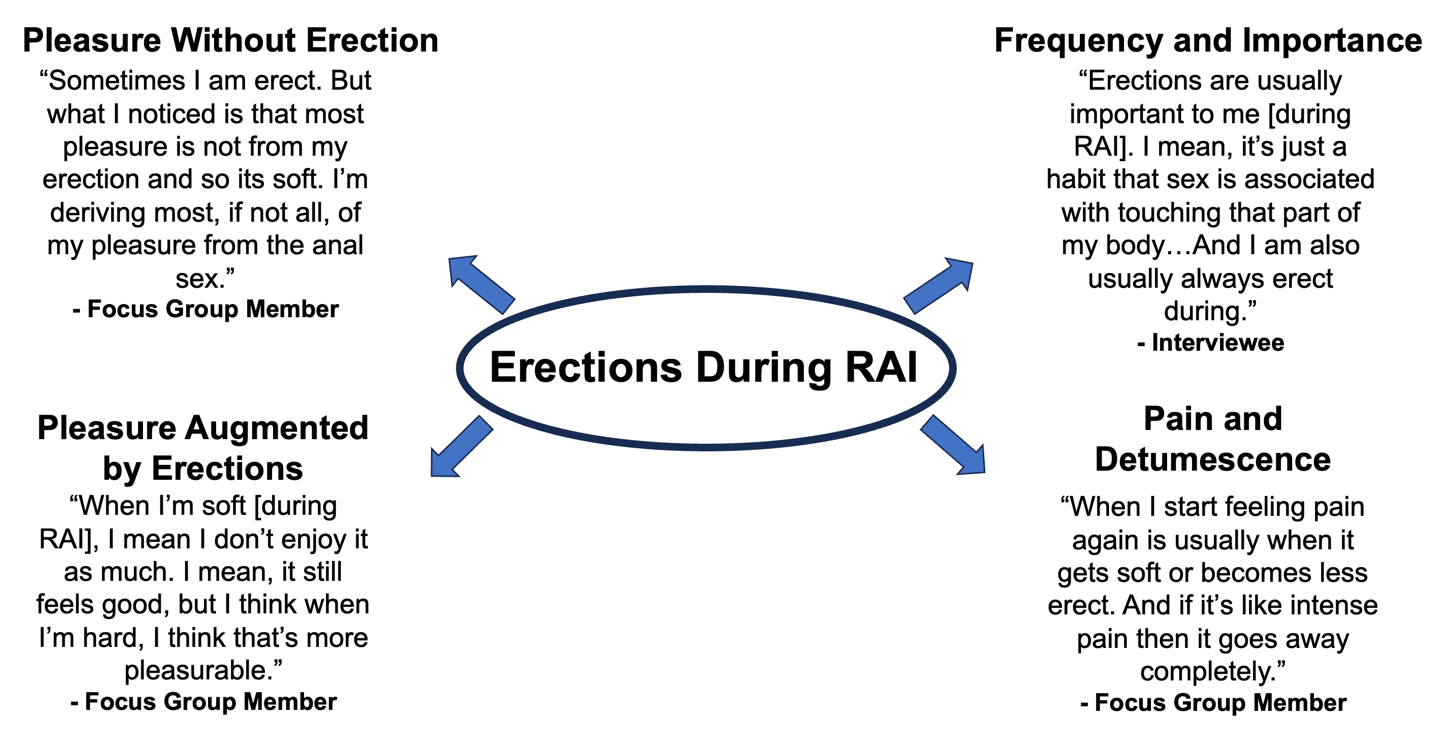


*The research methodology for this qualitative dataset is published (Gaither et al, 2023), reference 2
