# Supplementary material for: Erectile dysfunction during receptive anal intercourse: an overlooked entity?
Source: J Sex Med. Author manuscript; Available in PMC 2026 Jan 14. (PMC12802361; doi:10.1093/jsxmed/qdaf126)
Supplement: Table1 [file NIHMS2136089-supplement-Table1.docx]

Supplemental Table 1. Demographics and sexual characteristics of the sample, 2022-2023

|  | n = 1040 |
| --- | --- |
| **Age, mean (standard deviation)** | 36.1 (12.6) |
| **Sexual orientation, n(%)*** |  |
| Straight | 46 (5) |
| Gay | 825 (82) |
| Bisexual/Pansexual | 111 (11) |
| Queer | 28 (3) |
| **Race/Ethnicity, n(%)*** |  |
| Asian/Pacific Islander | 77 (8) |
| Black or African American | 34 (3) |
| Hispanic/Latino/Spanish | 107 (11) |
| Native American/Alaska Native | 4 (0.4) |
| White | 720 (71) |
| Multiracial | 27 (3) |
| Other | 43 (4) |
| **Location, n(%)*** |  |
| Africa | 22 (2) |
| Asia | 24 (2) |
| Australia | 38 (4) |
| Europe | 207 (20) |
| North America | 681 (67) |
| South America | 40 (4) |
| **Relationship Status, n(%)*** |  |
| Single | 359 (36) |
| Closed relationship (monogamous) | 402 (40) |
| Open relationship (polyamorous) | 249 (25) |
| **Age at first RAI, mean (SD)** | 21 (6.8) |
| **Sexual frequency, n(%)*** |  |
| Daily | 55 (5) |
| A few times a week | 258 (25) |
| Weekly | 349 (34) |
| Monthly | 207 (20) |
| Less than monthly | 145 (14) |
| **Number of sexual partners in the past 6 months, n(%)*** |  |
| 0 | 11 (1) |
| 1 | 405 (40) |
| 2-5 | 337 (33) |
| 6-10 | 129 (13) |
| >10 | 130 (13) |
| **Lifetime bottoming experiences, n(%)*** |  |
| <10 | 82 (8) |
| 11-15 | 261 (26) |
| 51-200 | 321 (32) |
| 201-500 | 172 (17) |
| >500 | 176 (17) |

*Missing data excluded
