# Supplementary material for: Erectile dysfunction during receptive anal intercourse: an overlooked entity?
Source: J Sex Med. Author manuscript; Available in PMC 2026 Jan 14. (PMC12802361; doi:10.1093/jsxmed/qdaf126)
Supplement: Table3 [file NIHMS2136089-supplement-Table3.docx]

Supplemental Table 3. Erectile dysfunction during receptive anal intercourse and its associated factors, 2022-2023; **sensitivity analysis A**

|  | **Erectile Dysfunction^a^**  **(n=46)** | **No Erectile Dysfunction (n=992)** | ***p* value** |
| --- | --- | --- | --- |
| **Age, mean (SD)** | 33.4 (9.7) | 36.3 (12.7) | 0.15 |
| **Sexual frequency, n(%)** |  |  |  |
| Daily | 3 (7) | 52 (5) | 0.39 |
| A few times a week | 13 (28) | 244 (25) |  |
| Weekly | 20 (43) | 328 (34) |  |
| Monthly | 7 (15) | 200 (21) |  |
| Less than monthly | 3 (7) | 142 (15) |  |
| **RAI lifetime exposure, n(%)^b^** |  |  |  |
| <10 times | 6 (13) | 76 (8) | 0.14 |
| 11-50 times | 14 (30) | 247 (26) |  |
| 51-200 times | 17 (37) | 303 (31) |  |
| 201-500 times | 2 (4) | 169 (18) |  |
| >500 times | 7 (15) | 169 (18) |  |
| **SHIM score, median (IQR)^c^** | 18 (12-21) | 20 (16-24) | 0.01 |
| **AUA-SI score, median (IQR)^d^** | 6 (2-17) | 5 (2-10) | 0.62 |
| **ASFI scores, median (IQR)^e^** |  |  |  |
| Pleasure | 14 (11-16) | 16 (13-18) | <0.01 |
| Pain | 18 (14-21) | 15 (12-19) | 0.01 |
| Urinary | 6.5 (5-8) | 6 (4-7) | 0.02 |
| Bowel | 10 (7-12) | 7 (5-10) | <0.01 |
| **Orgasm frequency, median (IQR)^f^** | 4 (3-4) | 4 (3-5) | 0.08 |
| **BSI, median (IQR)^g^** | 12 (4-24) | 5 (2-11) | 0.01 |
| **CPSI, median (IQR)^h^** | 5 (1-12) | 5 (2-10) | 0.96 |
| **Sexualized drug use, median (IQR)^f^** |  |  |  |
| Poppers | 2 (1-3) | 1 (1-3) | 0.22 |
| Alcohol | 2 (1-3) | 1 (1-3) | 0.47 |
| Marijuana | 1 (1-2) | 1 (1-2) | 0.26 |
| Methamphetamine | 1 (1-1) | 1 (1-1) | 0.01 |
| Nicotine | 1 (1-3) | 1 (1-1) | <0.01 |

a- ED is defined as rarely or never experiencing an erection during RAI with very or extreme bother

b- Approximate number of times the person has engaged in RAI within their lifetime

c- SHIM- Sexual Health Inventory for Men

d- AUA-SI- American Urologic Association- symptom index

e- ASFI- Anorectal Sexual Function Index (Gaither et al, 2024 in Urology)

f- Measured on 5-point Likert scale (1-never, 2-rarely, 3-sometimes, 4-often, 5-always)

g- BSI- Brief Symptom Inventory, assessment of mental health symptoms

h- CPSI- chronic prostatitis symptom index
