# Supplementary material for: Erectile dysfunction during receptive anal intercourse: an overlooked entity?
Source: J Sex Med. Author manuscript; Available in PMC 2026 Jan 14. (PMC12802361; doi:10.1093/jsxmed/qdaf126)
Supplement: Table2 [file NIHMS2136089-supplement-Table2.docx]

Supplemental Table 2. Erectile dysfunction during receptive anal intercourse and its associated factors, 2022-2023.

|  | **Erectile Dysfunction^a^**  **(n=213)** | **No Erectile Dysfunction**  **(n=827)** | ***p* value** |
| --- | --- | --- | --- |
| **Age, mean (SD)** | 35.7 (11.8) | 36.2 (12.7) | 0.58 |
| **Sexual frequency, n(%)** |  |  |  |
| Daily | 18 (9) | 37 (5) | 0.06 |
| A few times a week | 59 (28) | 199 (25) |  |
| Weekly | 66 (32) | 283 (35) |  |
| Monthly | 44 (21) | 163 (20) |  |
| Less than monthly | 22 (11) | 123 (15) |  |
| **RAI lifetime exposure, n(%)^b^** |  |  |  |
| <10 times | 13 (6) | 69 (9) | 0.27 |
| 11-50 times | 61 (29) | 200 (25) |  |
| 51-200 times | 63 (30) | 258 (32) |  |
| 201-500 times | 29 (14) | 143 (18) |  |
| >500 times | 42 (20) | 134 (17) |  |
| **SHIM score, median (IQR)^c^** | 18 (13-22) | 20 (16-24) | <0.01 |
| **AUA-SI score, median (IQR)^d^** | 6 (2-13) | 5 (2-9) | 0.44 |
| **ASFI scores, median (IQR)^e^** |  |  |  |
| Pleasure | 15 (12-17) | 16 (14-18) | <0.01 |
| Pain | 17 (13-21) | 15 (12-19) | <0.01 |
| Urinary | 7 (5-9) | 5 (4-7) | <0.01 |
| Bowel | 9 (6-12) | 7 (5-10) | <0.01 |
| **Orgasm frequency, median (IQR)^f^** | 4 (3-4) | 4 (3-5) | 0.02 |
| **BSI, median (IQR)^g^** | 6 (2-15) | 5 (2-11) | 0.17 |
| **CPSI, median (IQR)^h^** | 5 (2-12) | 5 (2-10) | 0.38 |
| **Sexualized drug use, median (IQR)^f^** |  |  |  |
| Poppers | 2 (1-3) | 1 (1-3) | 0.09 |
| Alcohol | 1 (1-3) | 1 (1-3) | 0.62 |
| Marijuana | 1 (1-2) | 1 (1-2) | 0.67 |
| Methamphetamine | 1 (1-1) | 1 (1-1) | <0.01 |
| Nicotine | 1 (1-2) | 1 (1-1) | <0.01 |

a- ED is defined as sometimes, rarely, or never experiencing an erection during RAI with moderate, very, or extreme bother

b- Approximate number of times the person has engaged in RAI within their lifetime

c- SHIM- Sexual Health Inventory for Men

d- AUA-SI- American Urologic Association- symptom index

e- ASFI- Anorectal Sexual Function Index (Gaither et al, 2024 in Urology)

f- Measured on 5-point Likert scale (1-never, 2-rarely, 3-sometimes, 4-often, 5-always)

g- BSI- Brief Symptom Inventory, assessment of mental health symptoms

h- CPSI- chronic prostatitis symptom index
